# Supplementary material for: A retinoic acid-dependent stroma-leukemia crosstalk promotes chronic lymphocytic leukemia progression
Source: Nat Commun. 2018 May 3;9:1787. doi: 10.1038/s41467-018-04150-7 (PMC5934403; doi:10.1038/s41467-018-04150-7)
Supplement: Supplementary file 1 — Supplementary Information [file 41467_2018_4150_MOESM1_ESM.pdf]

A Retinoic Acid-dependent Stroma-Leukemia Crosstalk Promotes Chronic Lymphocytic Leukemia Progression

Farinello et al.

Supplementary table 1: primary antibodies for immunofluorescence and FACS

**Antibody**	**Species**	**Conjugation**	**Company**	**Dilution**	**Clone / Cat. no.**
CD19	Rat	PE	BD pharmingen	1:50 for FACS	Clone 1D3 / Cat. no. 553786
CD5	Rat	APC	BD pharmingen	1:50 for FACS	Clone 53-7.3 / Cat. no. 550035
CD35	Rat	Biotinylated	BD pharmingen	1:100	Clone 8C12 / Cat. no. 553816
Collagen IV	Rabbit	-	Abcam	1:500	Cat. no. Ab19808
CXCL13 (for mouse)	Goat	-	R&D systems	1:100	Cat. no. AF470
Nidogen 2	Rabbit	-	Abcam	1:500	Cat. no. Ab14513
Laminin	Rabbit	-	Sigma Aldrich	1:300	Cat. no. L9393
MadCAM-1	Rat	Biotinylated	BD pharmingen	1:50	Clone MECA-367 / Cat. no. 553808
MOMA-1	Rat	Biotinylated	Abcam	1:500	Cat. no. Ab51814
CD21/35	Rabbit	PE	eBioscience	1:100	Clone 4E3/ Cat. no. 12-0212
GFP	Rabbit	-	Abcam	1:500	Cat. no. ab13970
CXCL13 (for human)	Goat	-	R&D systems	1:30	Cat. no. AF801
IgD (for human)	Goat	Biotinylated	SouthernBiotech	1:100	Cat. no. 2030-08
MAdCAM (for human)	Mouse	-	Serotech	1:100	Clone 314G8 Cat. no.

Supplementary table 2: secondary reagents

Antigen	Fluorochrome	Company	Dilution	Cat. no.
Anti-goat	Biotinylated	Vector	1:500	BA-9500
Anti-rabbit	Alexa Fluor 546	Invitrogen	1:500	A-11035
Anti-mouse	Alexa Fluor 568	Invitrogen	1:500	A-11031
Anti-rabbit	Alexa Fluor 488	Invitrogen	1:500	A-21441
Anti-rabbit	Alexa Fluor 647	Invitrogen	1:500	A-31573
Anti-rat	Alexa Fluor 488	Invitrogen	1:500	A-21208
Streptavidin	HRP	Perkin Elmer	1:500	NEL750
Streptavidin	Alexa Fluor 546	Invitrogen	1:500	S11225
Streptavidin	Alexa Fluor 488	Invitrogen	1:500	S11223
Streptavidin	Alexa Fluor 647	Invitrogen	1:500	S21374

Supplementary table 3: Sequence of Specific Primers and Probes Used for qPCR Analysis (Probe number corresponds to Universal Probe Library – Roche)

Gene	Forward Sequence	Reverse Sequence	Probe N°	Species
Aldh1a1	GCTGAACAAGCTGGCTGAC	CCATTGAGTGCCTCCATTGTA	#84	M. Musculus
Atraid	TGAGGATACATCGAAGCTAATGC	AGGGAACAGTTCTGAAGATCTAGC	#27	M. Musculus
Col1a1	ACCTAAGGGTACCGCTGGA	GAGCTCCAGCTTCTCCATCTT	#18	M. Musculus
Col3a1	ACCTCCTGGTGCTCTTGGT	CACGCTCTCCAGGTCGTC	#58	M. Musculus
Col4a6	CAGCCTCTGGATCGGATACT	ACTAGGGACTGGCCTCCAC	#3	M. Musculus
Cyp1b1	GGAAACCACGCTTCATCG	AGGACGGAGAAGAGTAGCAGAA	#84	M. Musculus
Cxcl12	CCAAACTGTGCCCTTCAGAT	CTTTAGCTTCGGGTCAATGC	#41	M. Musculus
Cxcl13	TGAGGCTCAGCACAGCAA	ATGGGCTTCCAGAATACCG	#84	M. Musculus
Itga1	GACTGACGTGACCATTGGAG	TGGTCACTTTAACTACAGCCACA	#17	M. Musculus
Lama5	GAGTCTGTGCGAGCTGTGG	TCGCCAGACGGTACCAAG	#80	M. Musculus
Loxl2	AGCTTTTCTTCTGGGCAACC	CTCCATCCTTGTCCTGTGCT	#18	M. Musculus
Nidogen-2	TGAGGCAGCTATCTGCTACAAT	CTTCCGGAACCGTGTCAG	#64	M. Musculus
Prelp	CAGAAGAGTGCCCCAGAGTC	ATGCCCTCATGATCCAGGT	#91	M. Musculus
Rdh10	CTCCCTGGGACTGTTCAGC	TCGTGAAAACCCACAACTCC	#67	M. Musculus
Rarbeta	CACCGGCATACTGCTCAA	CAAACGAAGCAGGGCTTG	#63	M. Musculus
Rargamma (isoform2)	CGCCGGACTTGAGTCTTTT	GCTCTGTGTCTCCACCGATT	#104	M. Musculus
qSma	TCACCATTGGAAACGAACG	ATAGGTGGTTTCGTGGATGC	#11	M. Musculus
Vcam-1	TGGTGAAATGGAATCTGAACC	CCCAGATGGTGGTTTCCTT	#34	M. Musculus
Rpl13a	CCCTCCACCCTATGACAAGA	GTAGGCTTCAGCCGAACAAC	#63	M. Musculus
RXRalpha	AAGCGGATCCCACACTTCT	GAAGGAGGCGATGAGCAG	18	H. Sapiens
Gapdh	AGCCACATCGCTCAGACAC	GCCCAATACGACCAAATCC	60	H. Sapiens

Vgll4	Nid2	C3,
Bst2	Crim1	Cd59a
Ifit3	Agrn	Cd74
Isg15	Cyr61	Crim1
Aldh3b1	Gas6	F11r
Lgals3bp	Acta2	Gadd45g
Vamp5	Ogn	Hp
Psme1	Prelp	Ier3
Aldh1a1	Serpina3n	Il6st
Usp18	Serpinb6b	Lamb2
Cyp1b1	Plau	Rgs16
Actg2	Sema3f	Trex1

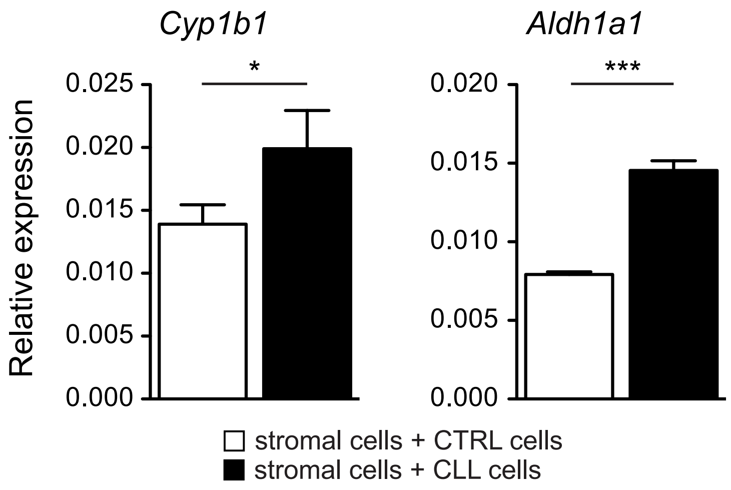

Supplementary Figure 1. Leukemic cells induce the expression of genes involved in RA-synthesis and tissue remodeling.
List of selected gene-signatures deregulated in stromal cells (mSSC) after the co-culture with murine *Eμ-TCL1* CLL or CTRL (splenic B cells) cells. In red are highlighted genes involved in RA synthesis, and Cyp1b1 and Aldh1a1 were validated by qPCR analysis. Data are from one out of four independent experiments with similar results. The mean of triplicates and ± SD are shown; * $p < 0.05$, *** $p < 0.001$.

Farinello et al., Figure S1

A

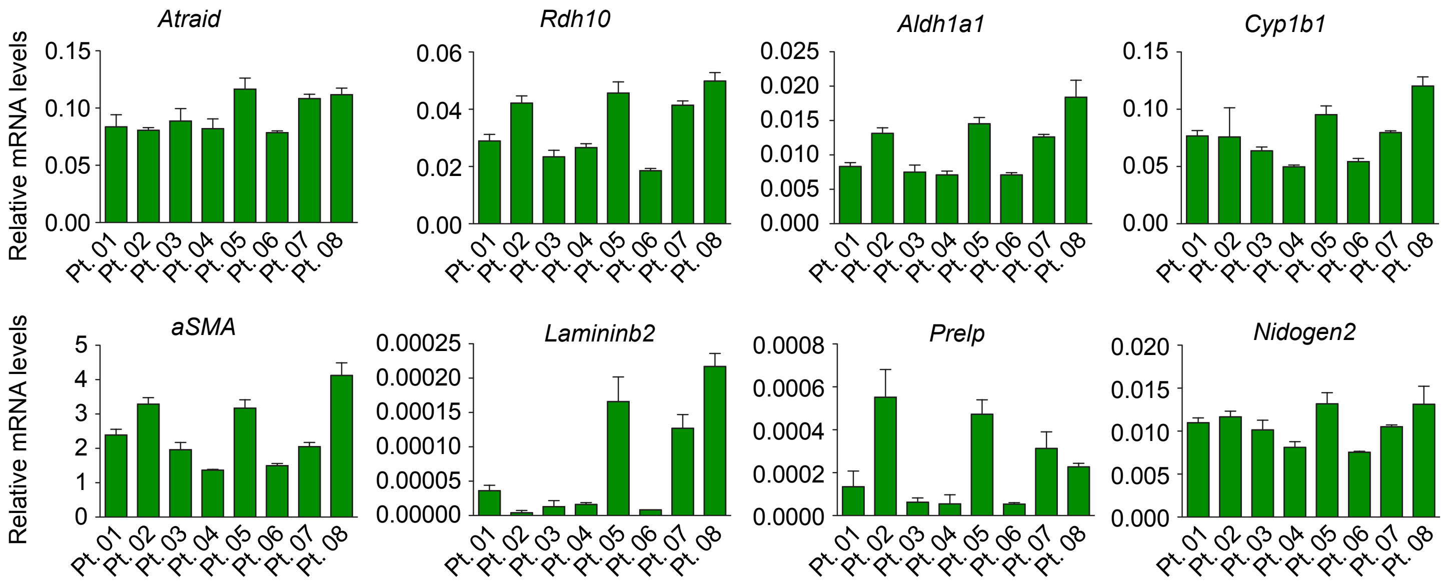

B

Paggetti et al. Blood 2015	Enrichment Significance (FDR)	CLL exosomes treated vs untreated BM-MSCs
Immune effector process	0,00E+00	upregulated genes
Regulation of immune system process	0,00E+00	
Immune response	0,00E+00	
Cytokine-mediated signaling pathway	0,00E+00	
Regulation of type I interferon production	0,00E+00	
Cytokine signaling in immune system	0,00E+00	
Interferon alpha/beta signaling	0,00E+00	
Interferon signaling	0,00E+00	
Cell cycle, Mitotic	1.75e-3	downregulated genes

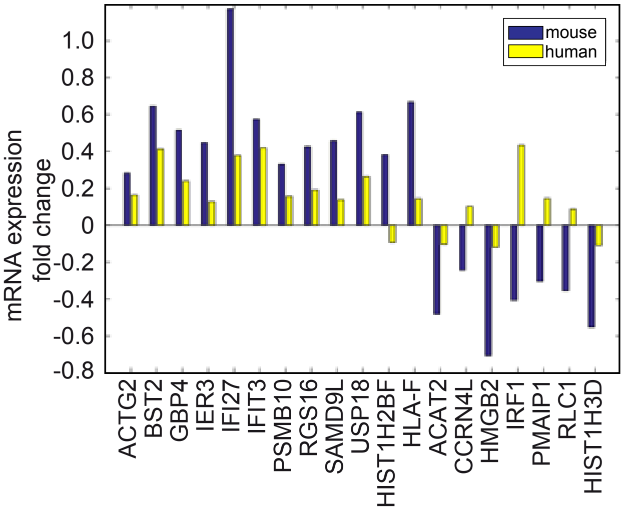

Supplementary Figure 2. Murine and human stroma-specific gene signature induced by human CLL.
A) qPCR analysis of stroma-specific genes from a murine stromal cell line (mSSC) cultured with human primary CLL cells for 24 hrs. B) Fold change comparison of common genes deregulated in stromal cells by mouse (our microarray data) and human CLL cells (from Paggetti et al. dataset).
Annotations of stromal-gene signatures by human CLL cells revealed similarities between mouse and human.

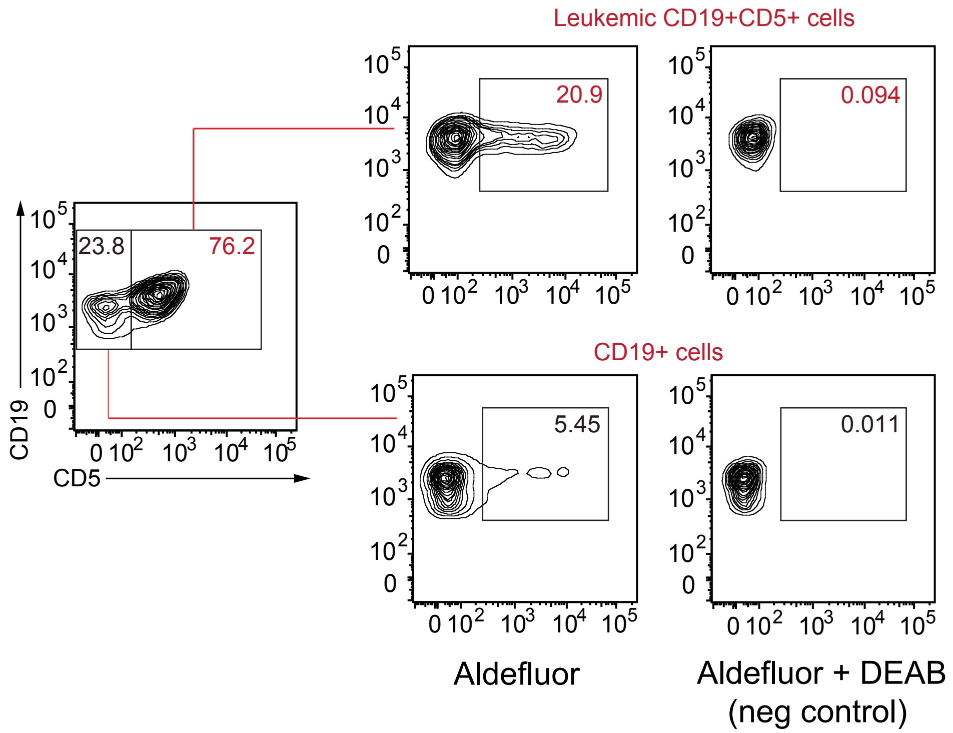

MOUSE	STRAIN	ALDEFLUOR+/CD19+	ALDEFLUOR+/CD19+ with DEAB	ALDEFLUOR+/CD19+CD5+	ALDEFLUOR+/CD19+CD5+ with DEAB
WT1	C57BL6	0.999	0.313	3.17	1.2
WT2	C57BL6	0.135	0.0215	0.532	0.233
TCL1 #1	*E*μ-*TCL1*	5.45	0.011	20.9	0.094
TCL1 #2	*E*μ-*TCL1*	14	1.21	30.3	0.52
TCL1 #3	*E*μ-*TCL1*	4.54	0.0807	10.2	0.0861
TCL1 #4	*E*μ-*TCL1*	8.93	0.0219	19.2	0.104
TCL1 #5	*E*μ-*TCL1*	15.6	1.2	17.5	0.3

Supplementary Figure 3. A fraction of leukemic cells possess ALDH activity.
*E*μ-*TCL1* leukemia cells isolated from the spleen of five different leukemia-bearing mice and two control mice were assessed for Aldefluor activity in the absence or presence of the ALDH control inhibitor (DEAB). One representative analysis is shown (highlighted row).

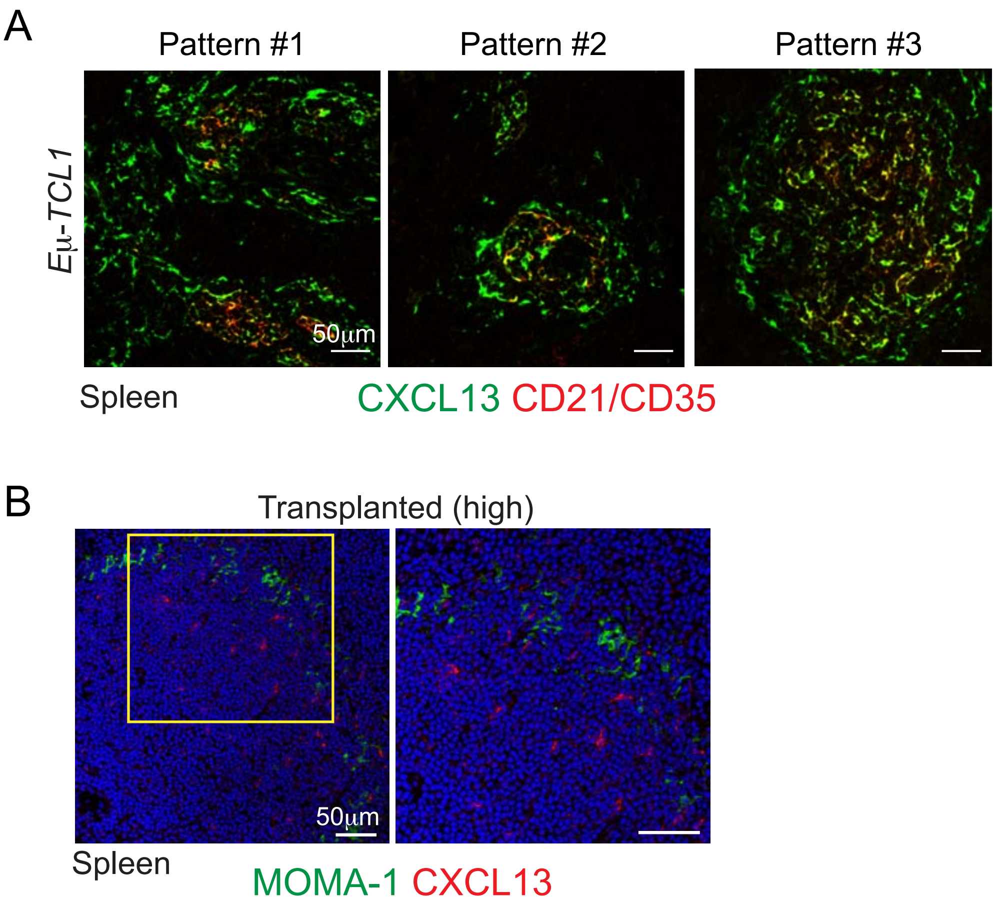

Supplementary Figure 4. Distribution of CXCL13 in spleen follicular stromal cells but not in macrophages. A) Confocal images of the spleen stained for CXCL13 (green) and CD21/CD35 (red) to visualize follicular stromal cells. B) Confocal image of the spleen from transplanted mice with high infiltration of leukemic cells stained for MOMA-1 (green) to visualize marginal metallophilic macrophages, and CXCL13 (red) to visualized reticular stromal cells. Data are representative of one out of five mice analyzed. Scale bars represent 50μm.

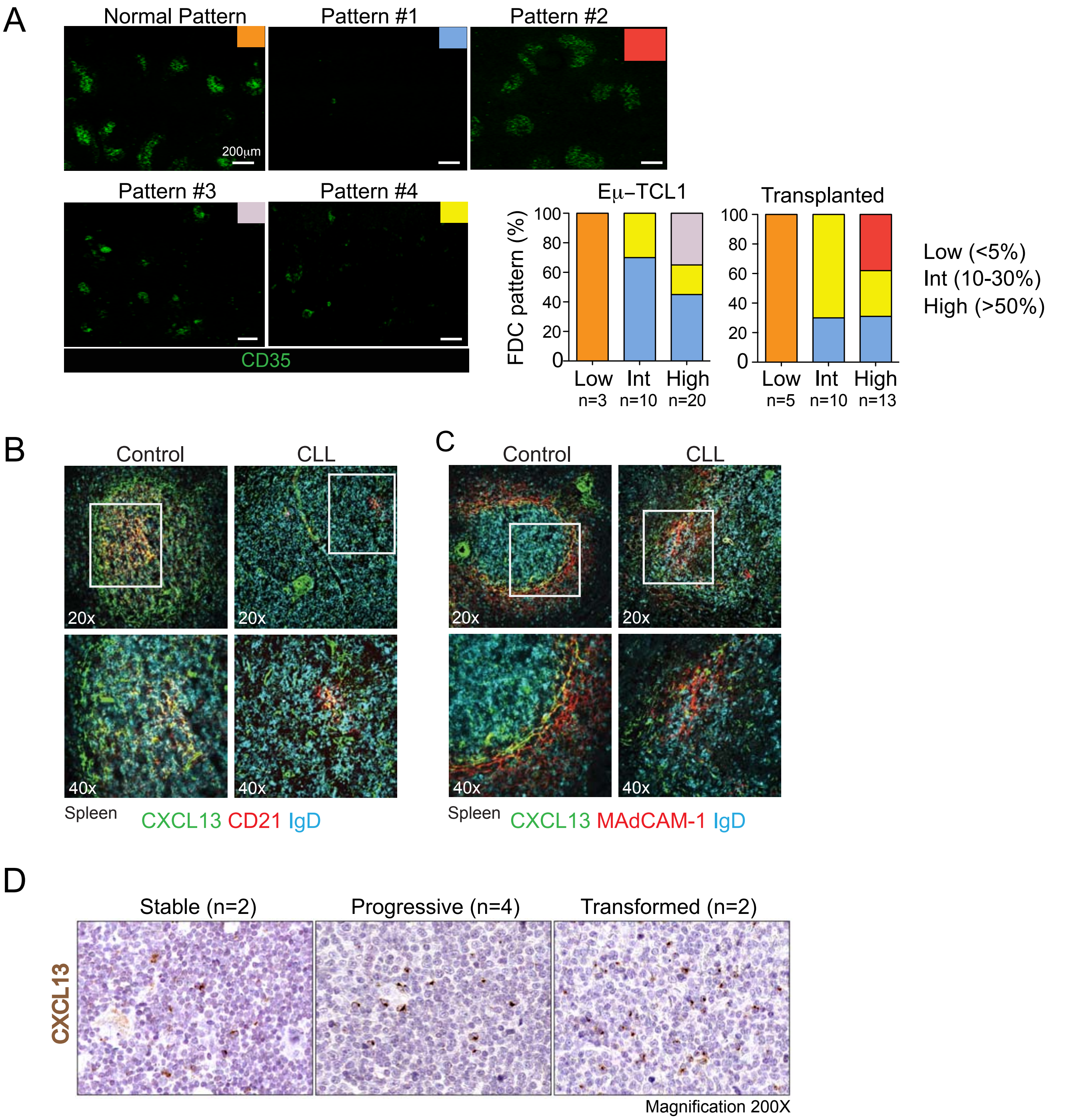

CXCL13+ cells in human CLL

		HPF						
Case number		1	2	3	4	5	#CXCL13+ cells	Mean value
1	Stable	68	80	46	69	118	76,2	61,1
2	Stable	60	94	20	14	42	46	
3	Progressive	25	26	23	26	24	24,8	32,4
4	Progressive	74	61	82	43	67	65,4	
5	Progressive	13	12	18	16	22	16,2	
6	Progressive	14	35	9	9	49	23,2	
7	Transformed	97	75	100	95	86	90,6	134,2
8	Transformed	192	162	190	220	125	177,8	

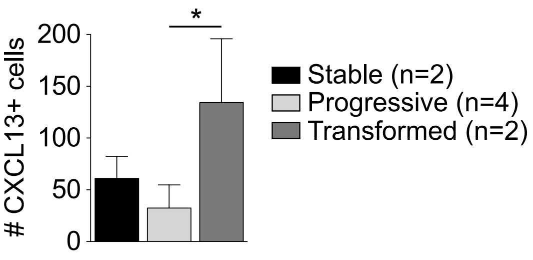

Supplementary Figure 5. Leukemia-stroma interactions cause disorganization of the follicular architecture. A) Representative confocal mosaic images of spleen sections. Tissues were isolated from transgenic and transplanted mice with low (<5%), intermediate (10-30%) and high (>50%) leukemia infiltration and stained for CD35 (green) to visualized FDCs. Graphs indicate the frequency of the different FDC patterns. Scale bars represent 200µm. B-C) Representative confocal images of human CLL spleens and controls stained for CXCL13 (green) and CD21 (red) and MAdCAM-1(red) to visualize FDC and MRC stromal cells respectively, and IgD (light blue) to visualize B cells. Original magnifications: 20X and 40x (insets). D) Bright field images of human CLL lymph node sections stained for CXCL13 (brown). Graph represents the average number of CXCL13+ cells from five high power fields (HPF) for each sample. Original magnification: 200x.

Farinello et al., Figure S5

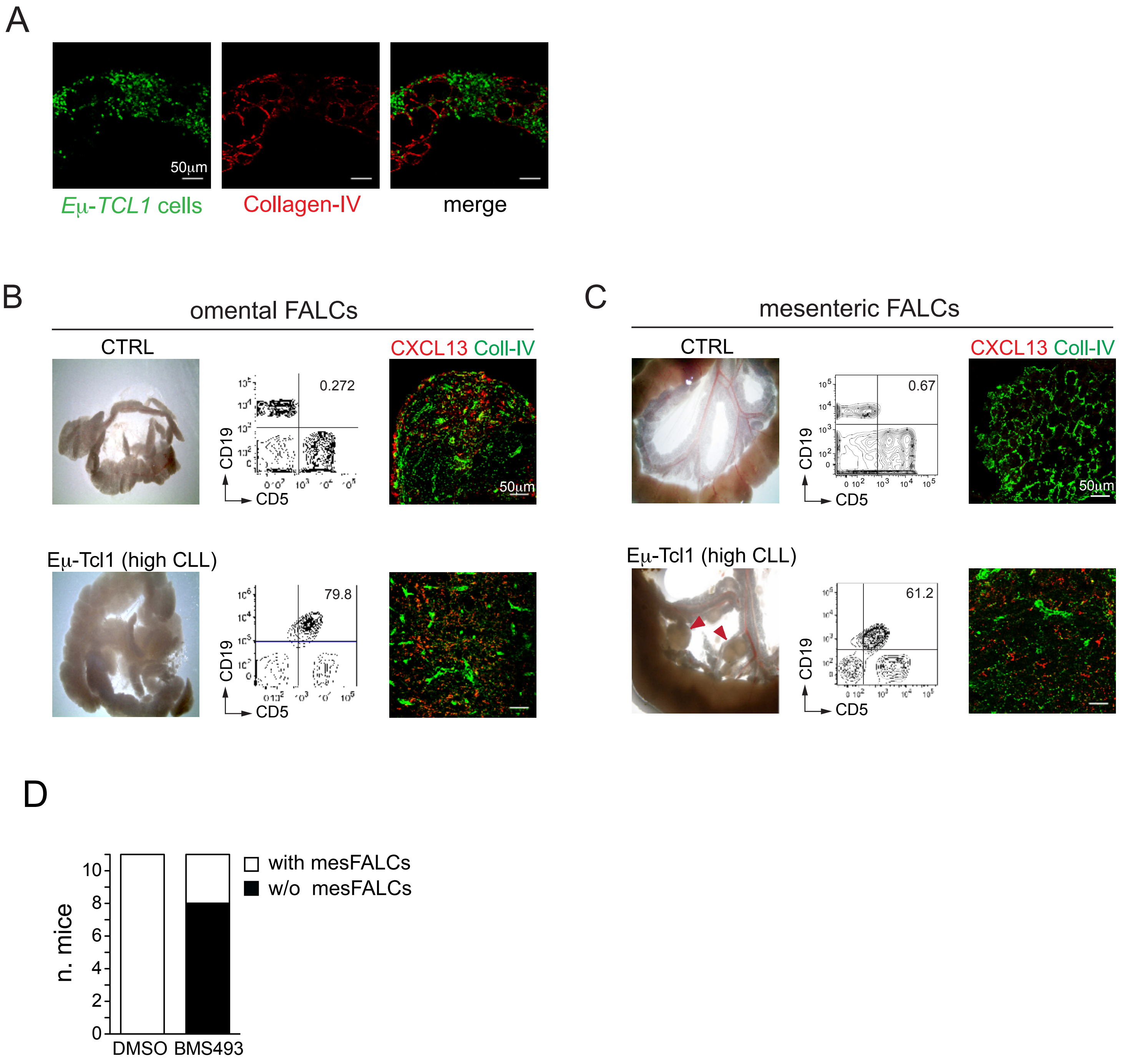

Supplementary Figure 6. Fat-associated lymphoid clusters support leukemia expansion and are inhibited by retinoid-antagonist therapy.
A) Representative confocal images of omental FALCs from wild type mice injected with CMFDA-labeled *Eμ-TCL1* CLL cells (green) and stained for collagen-IV (red). Scale bars represent 50μm. B-C) Representative bright-field images, flow cytometry plots showing CD5 and CD19 staining, and representative confocal images stained for CXCL13 (red) and Collagen-IV (green) from control or leukemic *Eμ-TCL1* mice with high percentage of leukemia in the blood. Scale bars represent 50μm. Data are representative of one out of five mice analyzed for each group. D) Number of mice treated with DMSO or BSM493 showing induction of mesenteric FALCs.
